# Molecular Etiology of Hearing Impairment in Inner Mongolia: mutations in *SLC26A4 *gene and relevant phenotype analysis

**DOI:** 10.1186/1479-5876-6-74

**Published:** 2008-11-30

**Authors:** Pu Dai, Yongyi Yuan, Deliang Huang, Xiuhui Zhu, Fei Yu, Dongyang Kang, Huijun Yuan, Bailin Wu, Dongyi Han, Lee-Jun C Wong

**Affiliations:** 1 Department of Otolaryngology and Genetic Testing Center for Deafness, Chinese PLA General Hospital, Beijing 100853, PR China; 2 Department of Otolaryngology, Chifeng Second Hospital, Chifeng City (Inner Mongolia), PR China; 3 Division of Genetics and Metabolism, Children's Hospital Boston, Harvard Medical School, Boston, Massachusetts, USA; 4 Department of Molecular and Human Genetics, Baylor College of Medicine, Houston, Texas, USA

## Abstract

**Background:**

The molecular etiology of hearing impairment in Chinese has not been thoroughly investigated. Study of *GJB2 *gene revealed that 30.4% of the patients with hearing loss in Inner Mongolia carried *GJB2 *mutations. The *SLC26A4 *gene mutations and relevant phenotype are analyzed in this study.

**Methods:**

One hundred and thirty-five deaf patients were included. The coding exons of *SLC26A4 *gene were sequence analyzed in 111 patients, not including 22 patients carrying bi-allelic *GJB2 *mutations or one patient carrying a known *GJB2 *dominant mutation as well as one patient with *mtDNA *1555A>G mutation. All patients with *SLC26A4 *mutations or variants were subjected to high resolution temporal bone CT scan and those with confirmed enlarged vestibular aqueduct and/or other inner ear malformation were then given further ultrasound scan of thyroid and thyroid hormone assays.

**Results:**

Twenty-six patients (19.26%, 26/135) were found carrying *SLC26A4 *mutation. Among them, 17 patients with bi-allelic *SLC26A4 *mutations were all confirmed to have EVA or other inner ear malformation by CT scan. Nine patients were heterozygous for one *SLC26A4 *mutation, including 3 confirmed to be EVA or EVA and Mondini dysplasia by CT scan. The most common mutation, IVS7-2A>G, accounted for 58.14% (25/43) of all *SLC26A4 *mutant alleles. The shape and function of thyroid were confirmed to be normal by thyroid ultrasound scan and thyroid hormone assays in 19 of the 20 patients with EVA or other inner ear malformation except one who had cystoid change in the right side of thyroid. No Pendred syndrome was diagnosed.

**Conclusion:**

In Inner Mongolia, China, mutations in *SLC26A4 *gene account for about 12.6% (17/135) of the patients with hearing loss. Together with *GJB2 *(23/135), *SLC26A4 *are the two most commonly mutated genes causing deafness in this region. Pendred syndrome is not detected in this deaf population. We established a new strategy that detects *SLC26A4 *mutations prior to the temporal bone CT scan to find EVA and inner ear malformation patients. This model has a unique advantage in epidemiologic study of large deaf population.

## Introduction

Every year in China, about 30,000 children, compared to 840 in UK and one of every one thousand infants in US, are born with congenital hearing impairment[1-3]. Hearing impairment is the most common neurosensory disorder in human that has an incidence of approximately 1 in 1000 children worldwide[4]. About 50–60% of these cases have a genetic cause. The most common molecular defects for nonsyndromic autosomal recessive deafness lie on Connexin 26, a gap junction protein encoded by the *GJB2*[5-12]. More than 150 mutations, polymorphisms and unclassified variants have been described in *GJB2 *to account for about 8–40% of molecular etiology of the patients with nonsyndromic hearing impairment [3]. However, about 80% of the patients with nonsyndromic hereditary deafness in China do not have mutations in *GJB2*[13].

Pendred syndrome (PS) is the most common form of syndromic deafness that accounts for about 10% of hereditary hearing impairment[14]. It is an autosomal recessive disorder caused by biallelic mutations in *SLC26A4 *resulting in hearing loss, enlargement of the vestibular aqueduct (EVA) and iodine organification defect in the thyroid gland[15]. EVA is always detected in the ears of patients with PS by computed tomography (CT) and magnetic resonance imaging (MRI)[16]. EVA is the most common form of the inner ear malformation associated with prelingual or postlingual sensorineural hearing loss and is an important feature of PS[17,18]. EVA may occur alone or in combination with an incomplete partition of the apical turn of the cochlea as part of Mondini deformity. PS is differentiated from nonsyndromic hearing loss with EVA by the presence of goiter, which usually develops later at around the time of puberty. Since environmental and other genetic factors may modulate the effects of *SLC26A4 *mutations on the development of goiter, the expression of goiter in PS patients is variable and may have incomplete penetrance[19]. *SLC26A4 *encodes an anion (chloride/iodide) transporter transmembrane protein, pendrin, which is expressed in the thyroid, kidney, and cochlea[20,21]. DNA sequence analysis identified more than 100 different mutations in *SLC26A4*[10,15,22-27]. The mutation spectrum varies widely among different ethnic groups[10,15,19,23,26-30]. Park and Pryor observed that patients with PS were always associated with two mutant alleles in *SLC26A4 *consistent with autosomal recessive disorder, whereas patients with nonsyndromic hearing loss and EVA might have one or zero mutant allele[15,19]. In Caucasian nonsyndromic EVA cohort, about one third of the patients had two mutant alleles, one third had one mutant allele and one third had zero[19]. In Japanese and Korean EVA patients, the proportion of patients having two identified mutant alleles in *SLC26A4 *is much higher, 57% and 81%, respectively[24,29]. Whereas in China, 97.9% EVA patients in simplex families were detected with either biallelic or monoallelic mutations, of which 88.4% were carrying biallelic variants and 9.5% with monoallelic mutation. Only 2.1% Chinese EVA patients had no mutant *SLC26A4 *allele detected[27]. In addition, the prevalent mutations in different ethnic groups are very different. Campbell et al. reported T416P and IVS8+1G>A as the two most frequent mutations in northern European population [22], while Blons et al. showed a completely different mutation spectrum that was extremely heterogeneous[23]. In Japanese, H723R accounted for 53% of the mutant alleles, and in Korean, the H723R and the IVS7-2A>G mutation was the most prevalent mutation accounting for 45.5% of patients with PS or EVA[19,29]. In China, IVS7-2A>G mutation was the most common form accounting for 57.63% of the mutant alleles[27]. All of the above studies focused on the EVA or Pendred syndrome patients.

In order to investigate the ratio of EVA or Pendred syndrome in Chinese hearing impairment patients and provide effective genetic testing and accurate counseling for hearing loss patients and families in China, we performed *SLC26A4 *sequence analysis in hearing impairment patients in Chifeng City from Inner Mongolia and then made a genotype-phenotype correlation analysis.

## Materials and methods

### Patients and DNA samples

A total of 135 deaf students from unrelated families of Chifeng Special Education School in Inner Mongolia, China, were included in this study. Among them, 73 patients suffered pre-lingual hearing impairment and 28 patients suffered post-lingual hearing impairment. The onset of deafness of 34 patients was unclear. Chifeng City Special Education School is the only deaf mute school in this area. All students with moderate to profound hearing loss from Chifeng city and within 500 km diameter of its neighboring area come to this school. This cohort of patients consists of 85 male and 50 female from 3 to 20 years old with the average age of 13.2 ± 3.6. The patients include 94 of Han, 31 of Mongolian, 7 of Man, and 3 of Hui races. This study was performed according to a protocol approved by the ethnicity committee of the Chinese PLA General Hospital. Informed consent was obtained from all parents prior to blood sampling. Parents were interviewed for age of onset, family history, mother's health condition during pregnancy and patient's clinical history including infection, possible head or brain injury and the usage of aminoglycoside antibiotics. In addition, 50 (race matched) controls with normal hearing were screened for *SLC26A4 *mutations by DHPLC followed by sequencing analysis. DNA was extracted from peripheral blood leukocytes using commercially available DNA extraction kit (Watson Biotechnologies Inc, Shanghai, China).

### Mutational analysis

DNA sequence analysis of *GJB2*, mitochondrial *12S rRNA *and *SLC26A4 *were performed by PCR amplification of the coding exons plus approximated 50–100 bp of the flanking intron regions followed by Big Dye sequencing and analysis using ABI 3100 DNA sequencing machine (ABI, Foster City, USA.) and ABI 3100 Analysis Software v.3.7 NT according to manufacturer's procedures. Patients with two *GJB2 *mutant alleles (22 cases) or one dominant mutant allele (one case) or *mtDNA *1555 A>G mutation (one case) were not further analyzed for *SLC26A4 *mutations. The exons of *SLC26A4 *of the remaining 111 patients were sequenced one by one starting from the frequently mutated exons until 2 mutant alleles were identified.

### CT scan and thyroid examination

Twenty-nine of 32 individuals who had mutations or variants in *SLC26A4 *were subjected to temporal bone computerized tomography (CT) scan for the diagnosis of EVA or inner ear malformation based on the criteria of a diameter of greater than 1.5 mm at the midpoint between the common crus and the external aperture[31]. To evaluate for Pendred syndrome, the ultrasound scan of thyroid and the thyroid hormone levels were measured in the patients positive for *SLC26A4 *mutations or variants. These procedures were performed at the Second Hospital of Chifeng City, Inner Mongolia, China. Ten patients with hyperthyroidism but normal hearing were enrolled as positive control for ultrasound scan of the thyroid and the levels of thyroid hormone. Since perchlorate discharge testing was not a general clinical practice in China, it was not used in this study.

## Results

All patients showed severe to profound bilateral sensorineural hearing impairment on audiograms except Patient 9 in Table 1 whose right ear pure tone average (PTA) is 55 dB.

### Correlation of genotype with age of onset of deafness

The average age of onset of patients with EVA and/or other inner ear malformation is 1.56 ± 1.23. The average age of onset of other patients is 0.97 ± 1.42. There is no significant statistic difference between the two groups (P value 0.09, t = 1.71). The average age of onset of patients with *SLC26A4 *mutations or variants is 1.27 ± 1.10. The average age of onset of patients without *SLC26A4 *mutations or variants is 1.03 ± 1.24. There is no significant statistic difference between the latter groups (P value 0.46, t = 0.727).

### SLC26A4 mutations

Sequence analysis of *SLC26A4 *in these 111 patients with hearing impairment identified 16 patients (1 to 16) with two confirmed pathogenic mutations (Table 1), and one (Patient 17) with compound heterozygote of two unclassified variants, Y375C and R470H, which are most likely pathogenic (Table 1). Six patients (19 to 24) carry one *SLC26A4 *mutant allele, and two patients (18 and 25) carry a novel unclassified missense variant, I491T and L597S, respectively, that are likely pathogenic due to their evolutionary conservation and conserved amino acid change. Patient 26 carried V659L, a pathogenic mutation that has also been found in a patient with EVA (Patient 11). The pathogenicity of V659L is reported by Wang et al. in Chinese enlarged vestibular aqueduct patients[27]. Each of patients 27 to 29 is heterozygous for an unclassified missense variant. Patients 27 and 28 carrying a single conserved amino acid change, I235V and T67S respectively, had normal vestibular aqueducts. These two missense variants are probably benign. The novel IVS12-6insT in Patient 29 does not predict a gain or loss of a spice site when analyzed using programe available on . So it is also considered benign. Thus, mutations in *SLC26A4 *were identified in 19.26% (26/135) patients with hearing impairment in Inner Mongolia, China, 17 with two mutant alleles and 9 with one mutant allele.

**Table 1:** Phenotype and genotype of *SLC26A4 *gene related hearing impairment in Inner mongilia

**Patient number**	**Age**	**Genotype**						**Phenotype**						
		**Allele 1**			**Allele 2**			**CT**	**Age of onset**	**Diameter (mm)**	**PTA (L) (dB)**	**PTA (R) (dB)**	**Thyroid hormone**	**US scan Of thyroid**
	
		Nucleotide Change	amino acid change	category	nucleotide change	amino acid change	category							

1	17	IVS7-2	aberrant splicing	pathogenic	IVS7-2	aberrant splicing	pathogenic	^a^EVA	0.7	3.28	82.	93	normal	normal
2	17	IVS7-2	aberrant splicing	pathogenic	IVS7-2	aberrant splicing	pathogenic	EVA	2	3.33	103	106	normal	normal
3	9	IVS7-2	aberrant splicing	pathogenic	IVS7-2	aberrant splicing	pathogenic	EVA	2.5	2.73	93	95	Total T3 slightly elevated	normal
4	16	IVS7-2	aberrant splicing	pathogenic	IVS7-2	aberrant splicing	pathogenic	EVA	0	2.73	97	97	normal	normal
5	10	IVS7-2	aberrant splicing	pathogenic	IVS7-2	aberrant splicing	pathogenic	EVA	1	3.64	76	93	normal	normal
6	14	IVS7-2	aberrant splicing	pathogenic	IVS7-2	aberrant splicing	pathogenic	EVA	2	2.73	96	83	normal	normal
7	10	IVS7-2	aberrant splicing	pathogenic	IVS7-2	aberrant splicing	pathogenic	EVA	1	2.0	88	95	normal	normal
8	8	IVS7-2	aberrant splicing	pathogenic	IVS7-2	aberrant splicing	pathogenic	EVA	2	1.64	101	95	normal	normal
9	19	IVS7-2	aberrant splicing	pathogenic	230A>T	K77I	pathogenic	EVA	4	2.22	71	55	normal	normal
10	16	IVS7-2	aberrant splicing	pathogenic	1229C>T	^b^T410M	pathogenic	EVA	3	4.55	78	77	normal	normal
11	14	IVS7-2	aberrant splicing	pathogenic	1975G>C	^b^V659L	pathogenic	EVA	3	4.19	95	95	normal	normal
12	13	IVS7-2	aberrant splicing	pathogenic	2168A>G	H723R	pathogenic	EVA	3.5	4.55	96	85	normal	normal
13	13	2168A>G	H723R	pathogenic	109G>T	E37X, nonsense mutation	pathogenic	EVA	0	2.89	90	87	normal	Cystoid change
14	19	2168A>G	H723R	pathogenic	1229C>T	^b^T410M	pathogenic	EVA	1.5	2.44	107	102	normal	normal
15	17	2168A>G	H723R	pathogenic	***2167C>G***	***H723D***	***Unclassified variant***	EVA	0.25	5.46	85	100	normal	normal
16	14	1173C>A	S391R	pathogenic	1229C>T	^b^T410M	pathogenic	EVA	0.1	3.33	95	90	normal	normal
17	10	***1124A>G***	***Y375C***	***Unclassified variant***	***1409G>A***	***R470H***	***Unclassified variant***	Vestibular and cochlear malformation	0.1		^a^NA	NA	NA	NA
18	19	***1472T>C***	***I491T***	***Unclassified variant***				EVA and Mondini	0.6	4.44	100	100	NA	NA
19	16	IVS7-2	aberrant splicing	pathogenic				EVA	2	5.46	93	92	Total T3 slightly elevated	normal
20	10	IVS7-2	aberrant splicing	pathogenic				EVA	2	2.66	76	77	normal	normal
21	17	IVS7-2	aberrant splicing	pathogenic	*1905G>A*	*E635E*	*Silent variant*	^a^ND	1		84	107	NA	NA
22	19	1174A>T	N392Y	pathogenic				ND	0		100	100	NA	NA
23	16	IVS7-2	aberrant splicing	pathogenic				^a^nl	1		110	102	NA	NA
24	24	IVS7-2	aberrant splicing	pathogenic				nl	1.1		100	100	NA	NA
25	19	***1790T>C***	***L597S***	***Unclassified variant***				nl	1.2		100	100	NA	NA
26	17	1975G>C	^b^V659L	pathogenic				nl	0		98	100	normal	normal
27	15	***757A>G***	***I253V***	***Unclassified variant***				nl	1		110	108	NA	NA
28	17	***200C>G***	***T67S***	***Unclassified variant***				nl	1.3		95	100	normal	normal
29	13	***IVS12-6 insT***	***Intron insertion***	***Unclassified variant***				nl	1		97	100	NA	NA
30	16	*225C>G*	*L75L*	*Silent variant*				ND	0		110	103	NA	NA
31	20	*678T>C*	*A226A*	*Silent variant*				nl	1		105	105	NA	NA
32	18	*1905G>A*	*E635E*	*Silent variant*				nl	0.7		110	110	normal	normal

Novel mutations are in bold and italic. nl = normal, EVA = enlarged vestibular aqueduct, ND = not determined, NA = not available, CT = computerized tomography, PTA(L) or (R) = pure tone average(left) or (R), IVS7 = intravening sequence 7 (intron 7), IVS12 = intravening sequence 12 (intron 12), Diameter = Diameter at the midpoint between the common crus and the external aperture.

A total of 7 different pathogenic mutations (IVS7-2A>G, E37X, K77I, S391R, N392Y, T410M, H723R) and 5 most likely pathogenic novel variants (Y375C, R470H, I491T, L597S, and H723D) were found. The E37X mutation that results in a premature stop codon and a truncated protein of less than 5% in length is predicted to be deleterious. The H723D mutation is caused by nucleotide substitution, c.2167C>G, which is predicted to be deleterious since a milder change at the same amino acid residue, H723R that has been found to be the most common pathogenic mutation in Japanese. Other missense mutations: K77I, S391R, N392Y, T410M and H723R have been reported in patients with hearing loss in other studies[26,27,29].

The most common mutation in our patient cohort is the aberrant splice site alteration, IVS7-2A>G. Eight patients were homozygotes, 4 patients were compound heterozygotes with another mutant allele, and 5 were heterozygotes without a second mutant allele. The IVS7-2A>G mutation accounts for 58.14% (25/43, counting only the definite pathogenic and most likely pathogenic variants) of all *SLC26A4 *mutant alleles (Table 1). These results suggest that a significant proportion (26/135 = 19.26%) of Chinese hearing impairment has molecular defects in *SLC26A4*.

### SLC26A4 mutations in control individuals

In order to determine carrier frequency in general population, *SLC26A4 *exons 2–21 of 50 normal hearing individuals were analyzed by DHPLC. One IVS7-2A>G heterozygote and one silent variant 2217A>G (Q739Q) were found. Although this control population is too small to reach the final conclusion, the carrier rate of *SLC26A4 *mutation in northern China is estimated to be about 2%. Polymorphisms in *SLC26A4 *gene appear to be rare in general population when compared to *GJB2 *gene.

### SLC26A4 polymorphisms

Three novel silent variants were identified; c.1905C>G (E635E), c.678T>C (A226A) and c.225C>G (L75L). These silent variants are not detected in the 50 control individuals.

### Comparison of SLC26A4 mutation spectrum in different patient population

In Asian population, more than 80% of nonsyndromic patients with EVA harbored mutations in *SLC26A4 *[19,27,29,30]. In Taiwan and China, both made up of >90% Han Chinese, the IVS7-2A>G splice mutation is the most prevalent. In Japan, H723R is the most prevalent. In Korea, IVS7-2A>G and H723R are the two most prevalent mutations. There seems to be a shift of mutation from IVS7-2A>G to H723R from China to Japan with Korea in the middle. Each population has its own rare variants that are not shared (Table 2). Mutations in *SLC26A4 *is very diverse in European and US populations without any prevalent mutations that account for more than 10% of the alleles in patients with Pendred syndrome or EVA (Table 2) [15,23,26]. Variants in *SLC26A4 *gene in Caucasians are rarely overlapped with those found in Asians.

**Table 2:** *SLC26A4 *mutation spectrum among different populations

	^a^Chinese	^a^Chinese	^a^Taiwanese	^a^Korean	^a^Japanese	^a^French	^a^Caucasian European	^a^US
^a^Total number of patients	135 NSHI (20 EVA)	95 EVA	38 EVA	26 EVA	10 PS + 32 EVA	30 PS	100 EVA	31 PS & EVA
Total mutant alleles identified	43 (100)	177(100)	57 (100)	45 (100)	57 (100)	50(100)	64 (100)	32 (100)
% of *SLC26A4 *mutation in total	15.92 (43/270)	93.16 (177/190)	75 (57/76)	86.5 (45/52)	67.86 (57/84)	83.33 (50/60)	32(64/200)	51.61(32/62)
IVS7-2A>G	**25 (62.5)**	**102(57.63)**	**48 (84.2)**	9 (20)	2 (3.51)			
T410M	3 (7.5)	4(2.26)	1 (1.75)			3 (6)	1(1.56)	
K77I	1 (2.5)	1(0.56)	1 (1.75)					
H723R	4 (10)	16(9.04)	1 (1.75)	**18 (40)**	**33 (57.9)**			
H723D	1 (2.5)							
S391R	1 (2.5)						1(1.56)	
N392Y	1 (2.5)	5(2.82)			1 (1.75)			
E37X	1 (2.5)	1(0.56)						
I491T	1 (2.5)							
Y375C	1 (2.5)							
R470H	1 (2.5)							
V659L	2(5)	1(0.56)						
S448L		1(0.56)	1 (1.75)					
T721M			1 (1.75)	1	3 (5.3)	1(2)	2(3.13)	
A372V			2 (3.51)		4 (7)			
A387V		1(0.56)	2 (3.51)					
2111ins5					2 (3.51)			
917delT					1 (1.75)			
1652insT					1 (1.75)			
IVS5-1G>A					1 (1.75)			
IVS8+1G>A					1 (1.75)	2(4)	3(4.69)	2(6.25)
322delC					1 (1.75)			
S610X					1 (1.75)			
C565Y					1 (1.75)			
K369E					1 (1.75)			
S657N					1 (1.75)			
S666F					1 (1.75)			
P123S					1 (1.75)			
M147V		2(1.13)		3 (6.67)	1 (1.75)			
IVS9+3A>G				4 (8.89)				
365insT				2 (4.44)				
S28R				1 (2.22)				
IVS4+4A>G				1 (2.22)				
P142R				1 (2.22)				
S166N				1 (2.22)				
G497S				1 (2.22)				
IVS14-1G>A		1(0.56)		1 (2.22)				
IVS15+5G>A		5(2.82)		1 (2.22)				
E625X				1 (2.22)				
L676Q		6(3.39)		1 (2.22)				
Y530H						**7(14)**	3(4.69)	1(3.13)
L445W						5(10)	**4(6.25)**	2(6.25)
IVS14+1G>A		1(0.56)				4(8)		
G209V						4(8)	1(1.56)	2(6.25)
T416P						3(6)		
L236P								2(6.25)
L597S	1 (2.5)							**4(12.5)**
P76L		1(0.56)						
T94I		3(1.69)						
P112S		1(0.56)						
349delC		1(0.56)						
387delC		1(0.56)						
G197R		1(0.56)						
G204V		1(0.56)						
D271G		1(0.56)						
916_917insG		2(1.13)						
G316X		1(0.56)						
N392S		1(0.56)						
1181_1183delTCT		1(0.56)						
R409H		3(1.69)						
Q421P		1(0.56)						
K440X		1(0.56)						
Q446X		1(0.56)						
S448X		1(0.56)						
Q514X		1(0.56)						
I529S		1(0.56)						
I532R		2(1.13)						
N558I		1(0.56)						
D573Y		1(0.56)						
1746delG		1(0.56)						
R685I		1(0.56)						

References	This study	(Wang et al. 2007)	(Wu et al. 2005)	(Park et al. 2004)	(Tsukamoto et al. 2003)	(Blons et al. 2004)	(Albert et al. 2006)	(Pryor et al. 2005)

Numbers in the parentheses are the percentages of mutant alleles in total SLC26A4 mutant alleles identified.

^a ^All mutations found in Asian populations are listed, Only the mutations that occurred in at least 3 unrelated families of the European and US populations or the mutations that had occurred in other populations are listed to show the diversity of mutations and the lack of prevalent mutations.

^b ^total number of chromosome studied = number of patients × 2

### Frequencies of SLC26A4 mutations in nonsyndromic deafness, EVA, and Pendred syndrome patients

CT scan was performed on 29 of the 32 patients listed in Table 1. Among them, 20 (69%) had EVA and/or Mondini dysplasia. Seventeen patients (17/20 = 85%) who harbored two mutations in *SLC26A4 *gene. had EVA, except one Patient (patient 17, Y375C and R470H) had vestibular and cochlea malformation. Only 3 out of the 7 patients who carry one heterozygous mutation had EVA, the other 4 were normal. All patients who were heterozygous for silent and most likely benign variants were normal on CT scan (Table 1). Since CT scan was performed after genotyping, only patients with *SLC26A4 *mutations or variants received CT scan. 100% of our patients with two mutant alleles (17/17) and only 33.3%(3/9) of patients with one mutant allele were confirmed to have EVA manifestation. The frequency of *SLC26A4 *mutations in our nonsyndromic deafness patients is 19.3% (26/135). Most reported studies focused on screening *SLC26A4 *mutations in the EVA or Pendred syndrome patients but not in the nonsyndromic deafness patients.

Other Asian studies report high frequency of finding *SLC26A4 *mutations in patients with EVA, 97.9, 87, 92, and 68% respectively for mainland China, Taiwanese, Korean and Japanese [8,27,29,32,33]. The mutation detection rate in Caucasian EVA patients is much lower, 53 and 40% respectively in UK and Europe [26,34]. In US population, mutations in *SLC26A4 *account for about one third of the nonsyndromic EVA patients [15]. Patients with Pendred syndrome, however, had higher mutation detection rate in *SLC26A4 *gene, 90% in a French study [23].

### CT scan

**Figure 1:**
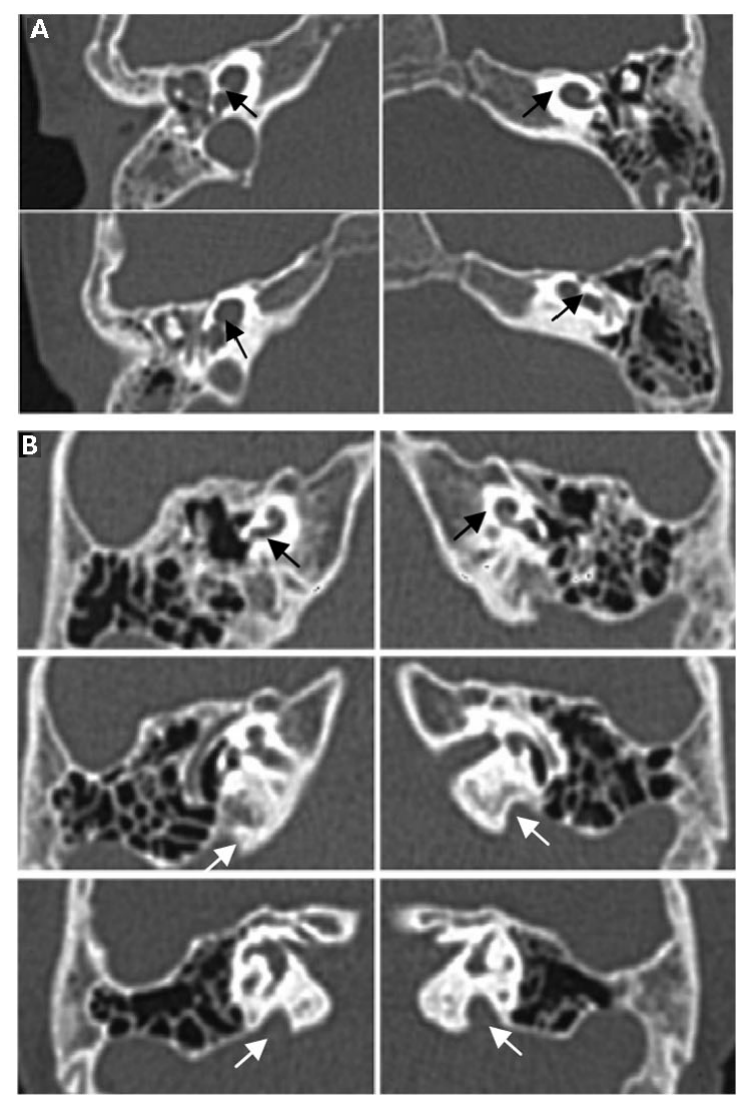
**A **1124A>G/1409G>A. (Patient 17). The black arrows in the CT picture showed the common cystic cavity of cochlea and vestibule. **B **1472T>C/wt. (Patient 18). The white arrows in the CT picture showed the hypolastic cochlea (Mondini). The black arrows in the CT picture showed EVA.

CT scan revealed EVA and/or other inner ear malformation in 20 patients. Sixteen patients (1 to16) had EVA and two pathogenic mutant alleles, consistent with autosomal recessive disorder caused by bi-allelic loss of function of pendrin protein (Table 1). Patient 17 had common cystic cavity of cochlea and vestibule without EVA. She carried two novel missense variants Y375C and R470H (Figure 1). Patient 18 had enlarged vestibular aqueduct with Mondini dysplasia (Figure 1). He carried a novel I491T variant. These results suggest that Y375C, R470H and I491T are most likely pathogenic. Two patients with one mutant IVS7-2A>G allele had EVA. CT scan results of Patients 21 and 22 (heterozygote IVS7-2A>G and N392Y respectively) were not available (Table 1). The remaining patients had normal CT scan. Testing of the 3 most frequent mutations, IVS7-2 A>G, H723R and T410M, can lead to finding 80% of patients with EVA or inner ear malformation in this cohort

Several patients have multiple affected siblings with the same two mutant alleles supporting that EVA is an autosomal recessive disease. For example, two sisters of patient 9 with the same genotype (IVS7-2A>G/K77I) and one sister of Patient 6 with homozygous IVS7-2A>G all have EVA. The parents of these two families are normal hearing individuals and carriers of corresponding *SLC26A4 *mutations.

### Thyroid ultrasound and thyroid hormone assays

Thyroid ultrasound was performed to determine presence or absence of goitre. None of the patients with *SLC26A4 *mutations or variants was diagnosed goitre. Only one patient (Patient 13) with EVA was found cystoid change in the thyroid by ultrasound scan, while there was no change in the thyroid hormone levels. Thyroid hormone assays showed that total T3 was slightly elevated in two patients (Patient 3 and Patient 19), but this abnormity had no clinical value when evaluated by endocrinologist from Chinese PLA General Hospital.

## Discussion

Diagnosis of Pendred syndrome EVA requires the evaluation of inner ear malformation by temporal bone CT scan. Unfortunately, in Chifeng City, Inner Mongolia, China, the temporal bone CT scan was too expensive to perform and there was lack of expertise for temporal bone evaluation. Under these circumstances, *SLC26A4 *mutation analysis may be the only alternative way for the diagnosis of EVA, since blood samples can be collected locally and sent elsewhere for DNA analysis. In this study, 100% patients (17/17) with bi-allelic mutation were confirmed to have EVA by CT scan performed in Chifeng Second Hospital with the help of a specialist from Beijing. Perchlorate discharge testing, a routine testing for thyroid function, is not available in most area of China. We use thyroid hormone testing and ultrasound scan of thyroid to examine the function and structure of thyroid instead. Our results indicate that none of patients have PS. These may be explained by a). testing methods were different, b). the age of patients undertaking thyroid ultrasound and thyroid hormone assays, 3 to 20, average 13.24 ± 3.92, in this study may be too young to have symptoms, c). phenotypic diversity due to different genetic background.

**Figure 2:**
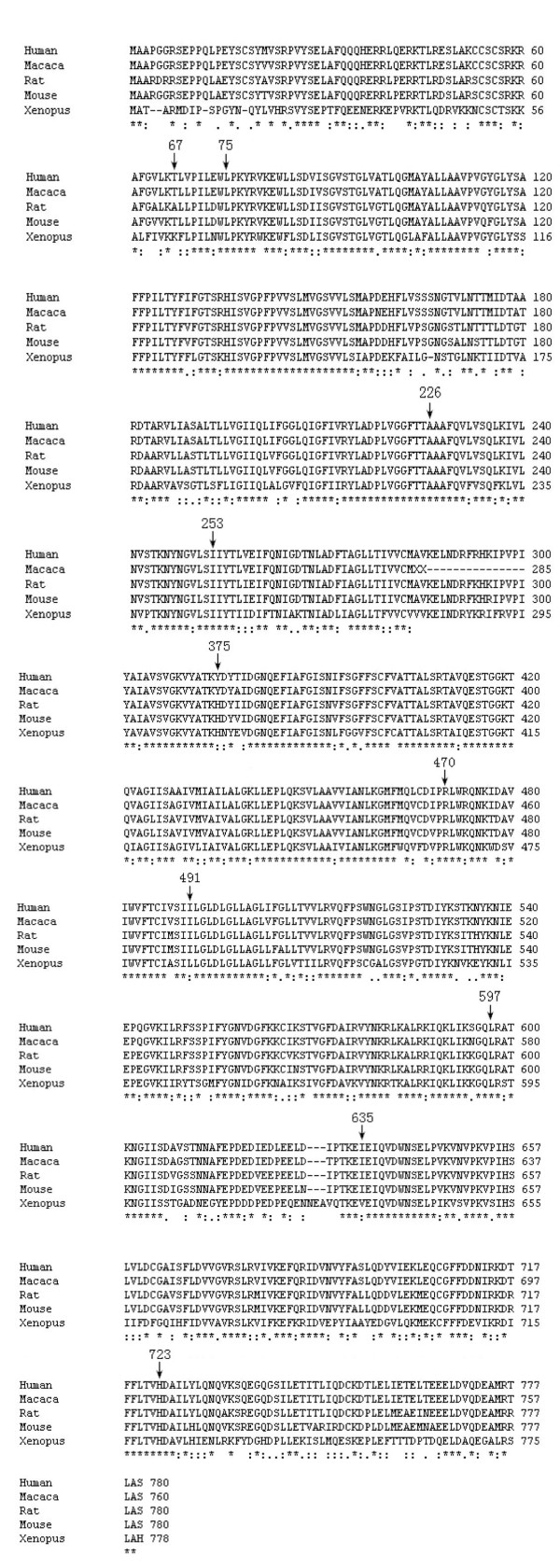
An alignment will display by default the following symbols denoting the degree of conservation observed in each column: "*" means that the residues or nucleotides in that column are identical in all sequences in the alignment. ":" means that conserved substitutions have been observed, "." means that semi-conserved substitutions are observed. The black arrows shows the amino acid related to newly found mutations or variants.

In this study, we found that *SLC26A4 *mutations were detected in nearly 20% of our patients with hearing impairment with IVS7-2A>G being the most prevalent mutation. Among the novel variants, Y375C, R470H, I491T, L597S and H723D were considered pathogenic based on a) they are located in evolutionarily conserved regions (Figure 2), b) substituted amino acids are structurally and functionally different from amino acids of the wild type, c) Y375C, R470H, I491T, L597S and H723D have been found in patients with EVA or other forms of inner ear malformation, and d) they were not present in our normal controls.

It's interesting to note that patient 18 with inner ear malformation carry one missense mutation only, whether the missense mutation causes dominant negative effect and/or specifies a different phenotype is not clear. Three patients (18 to 20) with EVA or other inner ear malformation harbored only one mutant allele. It's possible that the second mutant allele has not yet been identified due to a) mutations deep in introns or promoter regions that are not sequenced, b) intragenic exon deletions, c) mutations in genes other than *SLC26A4 *may involve in the pathogenesis (digenic). Thus, the mutations in the *SLC26A4 *gene account for at least 12.6% (17/135) of the patients with nonsyndromic hearing loss, making it as equally commonly mutated gene as *GJB2 *(23/135 no significant difference found after statistical analysis, P > 0.05) in patients from Inner Mongolia.

Unlike *GJB2 *which is a small gene with a lot of missence variants, *SLC26A4 *is a relatively large gene with rare missense benign polymorphisms or variants. Thus, novel missense variant in *SLC26A4 *is possibly pathogenic. Two questions were raised: can the autosomal recessive *SLC26A4 *mutations cause hearing impairment without EVA or other inner ear malformation, and are there other genes involved in the pathogenesis of hearing loss with *SLC26A4 *(digenic). To answer the first question, screening of the *SLC26A4 *mutations in a large NSHI population without EVA is necessary. For the second question, Malin Hulander reported that the lack of pendrin expression led to deafness and expansion of the endolymphatic compartment in inner ears of Foxi1 null mutant mice [34]. His observation provides the direct evidence that other genes may modulate the expression of *SLC26A4*. Alternatively there may be dominant negative effect.

The *SLC26A4 *mutation spectrum in ChiFeng City, Inner Mongolia is similar to that reported in Chinese population but different from that of Japanese. There is a gradient shift of the most prevalent mutation from IVS7-2A>G to H723R, respectively, from Chinese to Japanese with both mutations being equally prevalent in Korean. This observation suggests that IVS7-2A>G and H723R mutations may be the ancient mutations in China and Japan respectively. The unique rare mutations evolved more recently. A recent study of 100 unrelated patients with EVA in European Caucasians by Albert et al. revealed a diverse mutation spectrum without prevalent mutations and only 40 patients carried *SLC26A4 *mutations[26]. Our previous study on the prevalence of *GJB2 *mutations in Chinese patients with hearing impairment demonstrated that *GJB2 *mutations were detected in 30.4% of the patients in ChiFeng city. Together, approximately 49.63% (41+26/135) of patients with NSHI in ChiFeng city carried mutations in *GJB2 *or *SLC26A4 *gene. Whereas about 33.1% and 3.5% of European patients with NSHI carried mutations in *GJB2 *and *SLC26A4 *respectively, with a total of 36.6%, comparable to that in our patient group [35]. It is not clear why the mutations in *SLC26A4 *account for much lower percentage of patients with EVA in Caucasian patients. Presumably, other genetic factors and environmental factors are involved in the pathogenesis of EVA in Caucasians.

The striking spot of this study is that a new strategy that detects *SLC26A4 *mutations prior to the temporal bone CT scan to find EVA patients are established. In China, the cost of temporal CT scan is 200 to 300 RMB, because of the relatively high cost, it is not possible to perform CT scan in every hearing loss patient in molecular epidemiologic study to diagnose EVA. Since 97.9% of Chinese EVA patients carry *SLC26A4 *mutation [27], *SLC26A4 *mutation in hearing loss patients indicates a high possibility of EVA. This model presents unique advantage in epidemiologic study in large-scale deaf population to find EVA.

## Conclusion

In Inner Mongolia, China, mutations in *SLC26A4 *gene account for at least 12.6% (17/135) of the patients with nonsyndromic hearing loss. Pendred syndrome is not detected in the Inner Mongolia deaf population. We established a new strategy that detects *SLC26A4 *mutations prior to the temporal bone CT scan to find EVA and inner ear malformation patients. This model has a unique advantage in epidemiologic study of large deaf population.

## Competing interests

The authors declare that they have no competing interests.

## Authors' contributions

Pu Dai, Yongyi Yuan and Deliang Huang carried out the molecular genetic studies, participated in the sequence alignment and drafted the manuscript. Xiuhui Zhu carried out temporal CT scan and thyroid hormone assays. Dongyang Kang participated in the sequence alignment. Fei Yu and Huijun Yuan participated in the design of the study and performed the statistical analysis. Dongyi Han and Bailin Wu conceived of the study, and participated in its design and coordination and helped to draft the manuscript. Lee-Jun Wong reviewed and interpreted the results, drafted and revised the manuscript. All authors read and approved the final manuscript.

## References

[B1] Davis A, bamford J, wilson I, Ramkalawan T, Forshaw M, Wright S (1997). A critical review of the role of neonatal hearing screening in the detection of congenital hearing impairment. Health Technol Assess.

[B2] Brody JE (2000). Personal Health; Early Detection of Infant Deafness Is Vital. Quated by The New York Times-Health.

[B3] Dai P, Liu X, Yu F, Zhu Q, Yuan Y, Yang S, Sun Q, Yuan H, W Y, Huang D, Han D (2006). Molecular etiology of patients with nonsyndromic hearing loss from deaf-muta schools in 18 provinces of China. Chinese Journalof Otology.

[B4] Cohen MM, Gorlin RJ, Gorlin RJ, Toriello HV, Cohen MM Epidemiology, etiology and genetic patterns. Hereditary hearing loss and its snydromes.

[B5] Estivill X, Fortina P, Surrey S, Rabionet R, Melchionda S, D'Agruma L, Mansfield E, Rappaport E, Govea N, Mila M, Zelante L, Gasparini P (1998). Connexin-26 mutations in sporadic and inherited sensorineural deafness. Lancet.

[B6] Lench N, Houseman M, Newton V, Van Camp G, Mueller R (1998). Connexin-26 mutations in sporadic non-syndromal sensorineural deafness. Lancet.

[B7] Morell RJ, Kim HJ, Hood LJ, Goforth L, Friderici K, Fisher R, Van Camp G, Berlin CI, Oddoux C, Ostrer H, Keats B, Friedman TB (1998). Mutations in the connexin 26 gene (GJB2) among Ashkenazi Jews with nonsyndromic recessive deafness. N Engl J Med.

[B8] Park HJ, Hahn SH, Chun YM, Park K, Kim HN (2000). Connexin26 mutations associated with nonsyndromic hearing loss. Laryngoscope.

[B9] Rabionet R, Zelante L, Lopez-Bigas N, D'Agruma L, Melchionda S, Restagno G, Arbones ML, Gasparini P, Estivill X (2000). Molecularbasis of childhood deafness resulting from mutations in the GJB2 (connexin 26) gene. Hum Genet.

[B10] Wilcox SA, Saunders K, Osborn AH, Arnold A, Wunderlich J, Kelly T, Collins V, Wilcox LJ, McKinlay Gardner RJ, Kamarinos M, Cone-Wesson B, Williamson R, Dahl HH (2000). High frequency hearing loss correlated with mutations in the GJB2 gene. Hum Genet.

[B11] Gabriel H, Kupsch P, Sudendey J, Winterhager E, Jahnke K, Lautermann J (2001). Mutations in the connexin26/GJB2 gene are the most common event in nonsyndromic hearing loss among the German population. Hum Mutat.

[B12] Ohtsuka A, Yuge I, Kimura S, Namba A, Abe S, Van Laer L, Van Camp G, Usami S (2003). GJB2 deafness gene shows a specific spectrum of mutations in Japan, including a frequent founder mutation. Hum Genet.

[B13] Dai Pu, Yu Fei, Han Bing, Yuan Yongyi, Li Qi, Wang Guojian, Liu Xin, He Jia, Huang Deliang, Kang Dongyang, Zhang Xin, Yuan Huijun, Schmitt Eric, Han Dongyi, Wong Lee-Jun (2007). The prevalence of the 235delC GJB2 mutation in a Chinese deaf population. Genetics IN Medicine.

[B14] Reardon W, Coffey R, Phelps PD, Luxon LM, Stephens D, Kendall-Taylor P, Britton KE, Grossman A, Trembath R (1997). Pendred syndrome – 100 years of underascertainment?. QJM.

[B15] Pryor SP, Madeo AC, Reynolds JC, Sarlis NJ, Arnos KS, Nance WE, Yang Y, Zalewski CK, Brewer CC, Butman JA, Griffith AJ (2005). SLC26A4/PDS genotype-phenotype correlation in hearing loss with enlargement of the vestibular aqueduct (EVA): evidence that Pendred syndrome and nonsyndromic EVA are distinct clinical and genetic entities. J Med Genet.

[B16] Phelps PD, Coffey RA, Trembath RC, Luxon LM, Grossman AB, Britton KE, Kendall-Taylor P, Graham JM, Cadge BC, Stephens SG, Pembrey ME, Reardon W (1998). Radiological malformations of the ear in Pendred syndrome. Clin Radiol.

[B17] Valvassori GE, Clemis JD (1978). The large vestibular aqueduct syndrome. Laryngoscope.

[B18] Cremers CW, Admiraal RJ, Huygen PL, Bolder C, Everett LA, Joosten FB, Green ED, van Camp G, Otten BJ (1998). Progressive hearing loss, hypoplasia of the cochlea and widened vestibular aqueducts are very common features in Pendred's syndrome. Int J Pediatr Otorhinolaryngol.

[B19] Park HJ, Shaukat S, Liu XZ, Hahn SH, Naz S, Ghosh M, Kim HN, Moon SK, Abe S, Tukamoto K, Riazuddin S, Kabra M, Erdenetungalag R, Radnaabazar J, Khan S, Pandya A, Usami SI, Nance WE, Wilcox ER, Riazuddin S, Griffith AJ (2003). Origins and frequencies of SLC26A4 (PDS) mutations in east and south Asians: global implications for the epidemiology of deafness. J Med Genet.

[B20] Everett LA, Morsli H, Wu DK, Green ED (1999). Expression pattern of the mouse ortholog of the Pendred's syndrome gene (Pds) suggests a key role for pendrin in the inner ear. Proc Natl Acad Sci USA.

[B21] Royaux IE, Suzuki K, Mori A, Katoh R, Everett LA, Kohn LD, Green ED (2000). Pendrin, the protein encoded by the Pendred syndrome gene (PDS), is an apical porter of iodide in the thyroid and is regulated by thyroglobulin in FRTL-5 cells. Endocrinology.

[B22] Campbell C, Cucci RA, Prasad S, Green GE, Edeal JB, Galer CE, Karniski LP, Sheffield VC, Smith RJ (2001). Pendred syndrome, DFNB4, and PDS/SLC26A4 identification of eight novel mutations and possible genotype-phenotype correlations. Hum Mutat.

[B23] Blons H, Feldmann D, Duval V, Messaz O, Denoyelle F, Loundon N, Sergout-Allaoui A, Houang M, Duriez F, Lacombe D, Delobel B, Leman J, Catros H, Journel H, Drouin-Garraud V, Obstoy MF, Toutain A, Oden S, Toublanc JE, Couderc R, Petit C, Garabedian EN, Marlin S (2004). Screening of SLC26A4 (PDS) gene in Pendred's syndrome: a large spectrum of mutations in France and phenotypic heterogeneity. Clin Genet.

[B24] Park HJ, Lee SJ, Jin HS, Lee JO, Go SH, Jang HS, Moon SK, Lee SC, Chun YM, Lee HK, Choi JY, Jung SC, Griffith AJ, Koo SK (2004). Genetic basis of hearing loss associated with enlarged vestibular aqueducts in Koreans. Clin Genet.

[B25] Prasad S, Kolln KA, Cucci RA, Trembath RC, Van Camp G, Smith RJ (2004). Pendred syndrome and DFNB4-mutation screening of SLC26A4 by denaturing high-performance liquid chromatography and the identification of eleven novel mutations. Am J Med Genet A.

[B26] Albert S, Blons H, Jonard L, Feldmann D, Chauvin P, Loundon N, Sergent-Allaoui A, Houang M, Joannard A, Schmerber S, Delobel B, Leman J, Journel H, Catros H, Dollfus H, Eliot MM, David A, Calais C, Drouin-Garraud V, Obstoy MF, Tran Ba, Huy P, Lacombe D, Duriez F, Francannet C, Bitoun P, Petit C, Garabedian EN, Couderc R, Marlin S, Denoyelle F (2006). SLC26A4 gene is frequently involved in nonsyndromic hearing impairment with enlarged vestibular aqueduct in Caucasian populations. Eur J Hum Genet.

[B27] Wang QJ, Zhao YL, Rao SQ, Guo YF, Yuan H, Zong L, Guan J, Xu BC, Wang DY, Han MK, Lan L, Zhai SQ, Shen Y (2007). A distinct spectrum of SLC26A4 mutations in patients with enlarged vestibular aqueduct in China. Clin Genet.

[B28] Lopez-Bigas N, Melchionda S, de Cid R, Grifa A, Zelante L, Govea N, Arbones ML, Gasparini P, Estivill X (2001). Identification of five new mutations of PDS/SLC26A4 in Mediterranean families with hearing impairment. Hum Mutat.

[B29] Tsukamoto K, Suzuki H, Harada D, Namba A, Abe S, Usami S (2003). Distribution and frequencies of PDS (SLC26A4) mutations in Pendred syndrome and nonsyndromic hearing loss associated with enlarged vestibular aqueduct: a unique spectrum of mutations in Japanese. Eur J Hum Genet.

[B30] Wu CC, Yeh TH, Chen PJ, Hsu CJ (2005). Prevalent SLC26A4 mutations in patients with enlarged vestibular aqueduct and/or Mondini dysplasia: a unique spectrum of mutations in Taiwan, including a frequent founder mutation. Laryngoscope.

[B31] Mafee MF, Charletta D, Kumar A, Belmont H (1992). Largevestibular aqueduct syndrome and congenital sensorineural hearing loss. AJNR.

[B32] Hwa HL, Ko TM, Hsu CJ, Huang CH, Chiang YL, Oong JL, Chen CC, Hsu CK (2003). Mutation spectrum of the connexin 26 (GJB2) gene in Taiwanese patients with prelingual deafness. Genet Med.

[B33] Shi GZ, Gong LX, Xu XH, Nie WY, Lin Q, Qi YS (2004). GJB2 gene mutations in newborns with non-syndromic hearing impairment in Northern China. Hear Res.

[B34] Hulander M, Kiernan AE, Blomqvist SR, Carlsson P, Samuelsson EJ, Johansson BR, Steel KP, Enerbäck S (2003). Lack of pendrin expression leads to deafness and expansion of the endolymphatic compartment in inner ears of Foxi1 null mutant mice. Development.

[B35] Hutchin T, Coy NN, Conlon H, Telford E, Bromelow K, Blaydon D, Taylor G, Coghill E, Brown S, Trembath R, Liu XZ, Bitner-Glindzicz M, Mueller R (2005). Assessment of the genetic causes of recessive childhood nonsyndromic deafness in the UK – implications for genetic testing. Clin Genet.

